# Persistence and generalization of adaptive changes in auditory localization behavior following unilateral conductive hearing loss

**DOI:** 10.3389/fnins.2023.1067937

**Published:** 2023-02-01

**Authors:** Ana Sanchez Jimenez, Katherine J. Willard, Victoria M. Bajo, Andrew J. King, Fernando R. Nodal

**Affiliations:** Department of Physiology, Anatomy and Genetics, University of Oxford, Oxford, United Kingdom

**Keywords:** perceptual learning, training, plasticity, adaptation, spatial hearing, binaural, monaural spectral cues, ferret

## Abstract

**Introduction:**

Sound localization relies on the neural processing of binaural and monaural spatial cues generated by the physical properties of the head and body. Hearing loss in one ear compromises binaural computations, impairing the ability to localize sounds in the horizontal plane. With appropriate training, adult individuals can adapt to this binaural imbalance and largely recover their localization accuracy. However, it remains unclear how long this learning is retained or whether it generalizes to other stimuli.

**Methods:**

We trained ferrets to localize broadband noise bursts in quiet conditions and measured their initial head orienting responses and approach-to-target behavior. To evaluate the persistence of auditory spatial learning, we tested the sound localization performance of the animals over repeated periods of monaural earplugging that were interleaved with short or long periods of normal binaural hearing. To explore learning generalization to other stimulus types, we measured the localization accuracy before and after adaptation using different bandwidth stimuli presented against constant or amplitude-modulated background noise.

**Results:**

Retention of learning resulted in a smaller initial deficit when the same ear was occluded on subsequent occasions. Each time, the animals’ performance recovered with training to near pre-plug levels of localization accuracy. By contrast, switching the earplug to the contralateral ear resulted in less adaptation, indicating that the capacity to learn a new strategy for localizing sound is more limited if the animals have previously adapted to conductive hearing loss in the opposite ear. Moreover, the degree of adaptation to the training stimulus for individual animals was significantly correlated with the extent to which learning extended to untrained octave band target sounds presented in silence and to broadband targets presented in background noise, suggesting that adaptation and generalization go hand in hand.

**Conclusions:**

Together, these findings provide further evidence for plasticity in the weighting of monaural and binaural cues during adaptation to unilateral conductive hearing loss, and show that the training-dependent recovery in spatial hearing can generalize to more naturalistic listening conditions, so long as the target sounds provide sufficient spatial information.

## Introduction

Sensory experience plays a vital role in calibrating neural circuits in the brain, so that they can be optimized to the prevailing sensory conditions. The experience-dependent plasticity of the maturing brain enables the processing that takes place within those circuits to adjust to growth-related changes in sensory inputs and to be matched to the statistics of the environment during sensitive periods of development (Keating and King, 2013; Kumpik and King, 2019). Plasticity in later life allows perceptual skills to improve with practice and affords a capacity to compensate for altered inputs associated with sensory disorders (Irvine, 2018; Kumpik and King, 2019).

In the auditory domain, the recovery of sound localization accuracy in mammals experiencing a unilateral conductive hearing loss has been widely used as a model for training-dependent plasticity (Keating and King, 2015). Sound localization is achieved by the computation of binaural and monaural spatial cues that result from the way sounds interact with the head and external ears (Grothe et al., 2010). Interaural time differences (ITDs) and interaural level differences (ILDs) provide the primary basis for localizing sounds in the horizontal plane, and the relationship between these binaural cues and directions in space is altered by occluding one ear, resulting in much less accurate localization judgments. Previous work has shown that adult humans and ferrets can be trained to adapt to this monaural hearing perturbation, thereby substantially recovering their localization accuracy (Kacelnik et al., 2006; Kumpik et al., 2010; Keating et al., 2016). This entails plasticity in the way monaural and binaural cues are used to localize sound (Keating and King, 2015), which is dependent on the functional integrity of auditory cortical circuits (Bajo et al., 2010, 2019; Nodal et al., 2010).

Adaptation to monaural hearing loss can be achieved by reweighting the different localization cues—with greater reliance placed on the unchanged monaural spectral cues available at the open ear—or through compensatory changes in binaural cue sensitivity (Kacelnik et al., 2006; Kumpik et al., 2010; Keating et al., 2016; Zonooz and Van Opstal, 2019). In addition, if a fixed sound level is used, the localization responses of adult humans wearing an earplug in one ear become more dependent during training on spatially ambiguous monaural head-shadow cues (Zonooz and Van Opstal, 2019). Evidence for cue reweighting is provided by the absence of an aftereffect when the earplug is removed, i.e., post-plug localization performance is indistinguishable from the normal-hearing pre-plug condition (Kacelnik et al., 2006; Kumpik et al., 2010; Keating et al., 2016; Bajo et al., 2019; Zonooz and Van Opstal, 2019). Cue reweighting provides a particularly effective strategy for adapting to asymmetric hearing loss because this helps to maintain accurate sound localization under different hearing conditions.

Spatial cue reweighting is therefore thought to be context specific, disappearing when normal binaural inputs are again experienced following earplug removal, and may arise from changes in the relative reliability of different localization cues (Van Wanrooij and Van Opstal, 2007; Keating and King, 2015). However, the cortex-dependent learning induced by monaural occlusion appears to leave a memory trace that can be retrieved when the same ear is re-plugged (Bajo et al., 2019). It is not known how long this memory trace is retained or whether the previously learned strategy for adapting to unilateral conductive hearing loss is ear specific. This is likely to be important in clinical conditions, such as otitis media with effusion (Hogan et al., 1997), where recurrent hearing loss is often experienced.

The extent to which perceptual training generalizes to untrained stimuli determines its therapeutic value. Previous studies have assessed adaptation to unilateral conductive hearing loss by presenting target sounds against a silent background in order to maximize learning. However, more naturalistic listening conditions can change abruptly as we navigate our daily lives, as do the target sounds we need to attend to. Therefore, determining whether auditory spatial learning generalizes beyond the training stimulus and to more complex listening environments is crucial for designing effective training protocols as part of rehabilitation strategies for hearing-impaired individuals.

In this study, we address both the persistence and generalization of auditory adaptive learning. Our results show that the cue reweighting that occurs when adult ferrets are first trained with abnormal auditory spatial cues takes place more readily when subsequent periods of monaural occlusion are experienced, indicating that the effects of learning persist for at least several months. However, adaptation to conductive hearing loss in one ear appears to reduce the capacity of the auditory system to compensate for occlusion of the contralateral ear. We also show that the training-dependent recovery in localization accuracy does generalize to other acoustic conditions and that the degree of adaptation correlates with the generalization of learning.

## Materials and methods

### Animals

Eleven adult female pigmented ferrets (*Mustela putorius*), aged ∼6 months at the start of the study and sourced from Marshal BioResources (United Kingdom), were used. All procedures were approved by the Committee on Animal Care and Ethical Review at the University of Oxford and licensed by the Home Office under the Animals (Scientific Procedures) Act (1986).

### Hardware and training procedures

Ferrets were trained by positive reinforcement on an approach-to-target sound localization task, using water as the reward. During the testing periods, the animals were housed in enriched cages in groups of 2 or 3 individuals with *ad libitum* access to dry food and controlled access to water. They were usually tested twice daily, with each session typically lasting 20–30 min, and received water as a reward contingent on their performance on the sound localization task. The animals’ body weight and water intake during behavioral testing were monitored daily to ensure that a welfare threshold of 15% weight drop was not reached, which would otherwise have resulted in the temporary suspension of testing. If required, extra water in the form of pureed food was provided at the end of each day to meet the estimated daily need of 60 ml/kg (based on daily measurements made on ferrets with free access to water in the animal facility at the University of Oxford).

**FIGURE 1:**
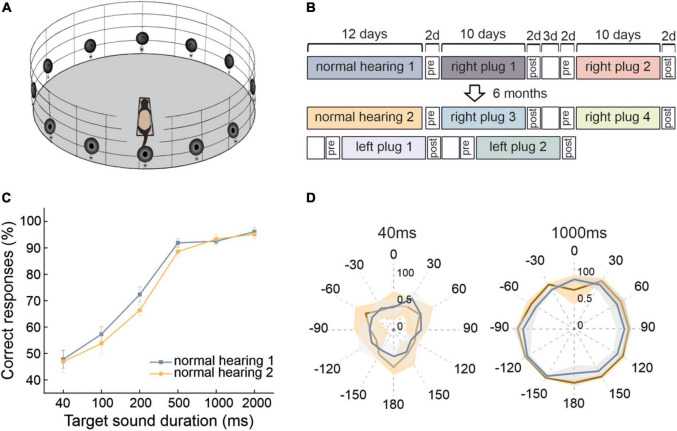
Stability of ferret sound localization performance over time when normal spatial cues are available. **(A)** Schematic of the circular testing arena with 12 loudspeakers and reward ports located at 30° intervals around the perimeter and a central platform on which the animal stands facing position 0° in order to trigger each stimulus presentation. **(B)** Timeline of behavioral testing to explore the persistence of adaptive learning over multiple periods of monaural occlusion interspersed by periods of normal binaural hearing. These animals were first trained to adapt with the right ear plugged and then with the left ear plugged. **(C)** Localization performance (percentage of correct trials averaged across all 12 target locations and sound levels) at different sound durations for two normal hearing periods that were separated by extensive localization training with one ear occluded. Error bars show the standard error of the mean. **(D)** Spatial distribution of the percentage correct scores from both normal hearing periods for the 40- and 1,000-ms broadband noise bursts. Lines represent the means and shaded areas the standard error of these scores across animals.

Behavioral testing was performed in a circular arena (Ø 140 cm) located inside a soundproof chamber (Figure 1A). The arena was equipped with 12 loudspeakers (FRS 8, Visaton, Crewe, United Kingdom) positioned at 30° intervals around its periphery in the horizontal plane. These loudspeakers were used to present target sounds for testing the ferrets’ sound localization accuracy, and an additional overhead speaker was located at the center of the chamber to provide background noise when required. Auditory stimuli were produced using MATLAB (MathWorks, United States) and presented using an RX8 multi I/O processor and two SA-8 power amplifiers (Tucker-Davies Technologies (TDT), Alachua, FL, United States). The output of each loudspeaker was digitally matched and flattened across sound frequencies.

Ferrets were trained to stand on a platform at the center of the arena and lick a waterspout facing the 0° position for a variable time, 300–500 ms, which triggered the presentation of a target sound stimulus from one of the 12 peripheral loudspeakers. An infrared beam at the back of the platform and a proximity infrared detector at the central waterspout were used to ensure the animal was positioned correctly when the stimulus was presented. The animal then had to signal its location by approaching the loudspeaker to obtain a water reward from its associated waterspout, which was equipped with an infrared detector and located just below the loudspeaker at the same azimuth. Only correct responses were rewarded (typically 150–250 μl of water depending on the animal and matched across all reward spouts). An incorrect response was followed by a “correction trial,” whereby the same sound was presented from the same location as in the previous unsuccessful trial. As in our previous work, up to two correction trials were allowed. If both correction trials elicited incorrect responses, an “easy trial” was triggered, in which the same sound was presented continuously until a response was made. Data from correction and easy trials were not included in the analysis.

We also obtained a more absolute measure of localization accuracy by tracking the ferret’s head movement using a reflective strip attached to the midline of the animal’s head. A high-speed camera located above the central platform captured the reflectance of the head-strip for 1 s from the onset of the target sound. The image acquisition rate was 60 frames per second (FPS) for the first part of the study (Imaging Source DMK21BF04) and 500 FPS for the second part of the study, following an upgrade to a high-speed camera (DMK 37AUX287).

### Experimental design

All animals were trained and tested on the approach-to-target sound localization task in a quiet background (hereafter referred to as “silence”) for 1–2 months until they showed a stable level of performance before the ear-plugging experiment started. Sound localization accuracy was measured using single bursts of broadband noise (low-pass cut-off frequency 30 kHz) in blocks of constant stimulus duration (2000, 1000, 500, 200, 100, and 40 ms). Within each block, we pseudorandomly varied both the stimulus location across the 12 loudspeakers and its level over a 56–84 dB SPL range in steps of 7 dB.

A reversible unilateral conductive hearing loss was induced by inserting an earplug (E-A-R Classic 3M) into the external auditory meatus and securing this in place with a silicone mold (Otoform KC, Dreve Otoplastik GmbH, Unna, Germany) placed in the concha of the external ear. Earplug insertion and removal were performed under sedation (medetomidine hydrochloride 0.1 mg/kg i.m., Domitor Orion Pharma, Reading, United Kingdom). An otoscopic examination was performed and a tympanogram (Kamplex KLT25 Audiometer, P.C. Werth, London, United Kingdom) was obtained both before insertion and after removal of an earplug to check the health status of the external and middle ear. No abnormalities were detected. Sedation was reversed with atipamezole hydrochloride (0.5 mg/kg s.c., Antisedan, Orion Pharma, Reading, United Kingdom). The acoustical effects of these earplugs have been characterized in previous studies in our laboratory in ferrets (Moore et al., 1989; Keating et al., 2013) and humans (Kumpik et al., 2010; Keating et al., 2016). We did not determine whether their properties changed during each period of monaural occlusion as this would have involved sedating the animals to remove and reposition the earplugs.

As in our previous work (Kacelnik et al., 2006; Bajo et al., 2010, 2019), we trained ferrets to adapt to an earplug using 1,000-ms long broadband noise bursts as the target stimuli on a background of silence. Individual ferrets wore an earplug in one ear for typically ≥10 days, until their performance stabilized over three consecutive days at a mean score of ≥70% correct across all 12 loudspeaker locations. We then removed the earplug and measured their localization accuracy again. We next examined either the persistence of adaptive learning by re-plugging the same ear and then the contralateral ear, or the generalization of learning using different target sounds and background noise combinations.

### Persistence of learning

To explore the persistence of the adaptive learning with one ear occluded, one group of 4 ferrets was subjected to several periods of unilateral conductive hearing loss interspersed within variable periods of normal binaural hearing.

Once their localization performance under normal hearing conditions had been measured using broadband noise bursts as target sounds in silence, a temporary unilateral conductive hearing loss was induced by inserting an earplug in the right ear (Figure 1B). Target sound levels and locations were pseudorandomized as described above. This first period of right ear occlusion (right plug 1) was followed by 7 days of normal hearing and then by another period of right ear occlusion (right plug 2) [data from right plug 1 and right plug 2 were reported in Bajo et al. (2019) as part of the control group in that study]. After a 6-month-long break in which the ferrets experienced normal hearing conditions, localization performance was measured during two further periods of right ear occlusion (right plug 3 and right plug 4). Finally, two periods of left ear occlusion were conducted (left plug 1 and left plug 2).

### Generalization of learning

A second group of 7 ferrets was used to explore whether the adaptive learning induced by training animals wearing an earplug to localize broadband noise target sounds on a silent background extended to other stimuli and acoustic conditions that were not available during training.

The sound localization performance of the animals was measured using different stimuli before, during and after occlusion of one ear. As before, adaptation to the earplug was achieved by training the animals to localize 1,000-ms broadband noise burst targets presented in silence, after which the animals were tested using different stimuli and background sounds with the earplug still in place. The target sound was either broadband (BB) noise (low-pass cutoff at 30 kHz) or one-octave wide narrowband noise (NB) centered on 16 kHz. The duration of the target sound was again 1,000 ms, and its level and location were pseudorandomly varied as before. These stimuli were presented either in silence (s) or in the presence of continuous background noise presented from an overhead loudspeaker. The background noise consisted of a broadband stimulus (low-pass cut-off at 30 kHz), whose envelope was either unmodulated (um) or amplitude modulated (am) at 5 Hz. The combination of these targets and backgrounds produced five different testing conditions: BBs, NBs, BBum, NBum, and NBam. The combination of broadband target sounds presented in an amplitude-modulated background (BBam) was discarded as a testing condition, because no difference was observed in localization accuracy between modulated and unmodulated backgrounds with this target sound in normal hearing conditions. Broadband target sounds were therefore only presented in silence (BBs) or with an unmodulated background (BBum).

### Role of dynamic and spectral cues

To explore the extent to which dynamic spatial cues that might be provided by the movement of the head during stimulus presentation and the spectral cues available at the non-occluded ear contribute to adaptation to unilateral conductive hearing loss, we included additional sessions during daily sound localization training with an earplug in place. Ferrets (*n* = 4) were trained to localize 1,000-ms broadband noise bursts in silence in the same fashion as described in previous sections. Every 2 days, we interspersed broadband and narrowband sounds (1/6-octave bandwidth centered at 15 kHz frequency to restrict the cues to ILDs) (Keating et al., 2015) at two different durations (200 and 1,000 ms), with each of these 4 stimulus types being presented with equal probability. The level of the broadband noise bursts was pseudorandomized as described previously, whereas the level of the narrowband stimuli was kept constant at 84 dB SPL, except for 10% of the trials in which it was 56 dB SPL. Incorrect responses to the broadband target sounds were followed by correction and easy trials, whereas no correction or easy trials were provided after an incorrect response on the narrowband trials. This was done to ensure that the animals did not learn a different strategy when localizing these stimuli that might have interfered with the normal adaptation process with broadband targets. However, all correct responses were rewarded to maintain the motivation of the animals.

### Data analysis

#### Head orienting responses

The angular position of the head-strip in each video frame was used to produce head movement traces. Trials in which <10% of consecutive frames contained measurable data were rejected. The start of a head turn was defined as a movement exceeding a threshold speed of 50 deg/s in the same direction (initial direction) over at least three consecutive frames. The timing of the last frame before a change in this initial direction was taken as the end of the head turn. The final head bearing was defined as the mean head angle calculated over the three frames before the end of the head movement. For the learning generalization experiment, traces were down-sampled to 50 FPS to make the data analysis comparable to the learning persistence experiment (Figure 1B), in which the sample rate was 60 FPS.

#### Statistical analysis

The rate of adaptation for each of the periods of monaural occlusion was computed by fitting regression lines to the percentage of correct responses across training day, with the shaded areas in the figures representing 95% confident intervals. The probability of making a correct response was compared across animals, hearing conditions (unilateral earplug or no earplug), and stimulus characteristics using a linear mixed model with a Bernoulli-distributed data, probit link function. Final head bearings were compared using repeated measures ANOVA. Data normality was checked using Q-Q plots and applying the Shapiro–Wilk test. All statistical tests were performed using RStudio (RStudio: Integrated Development for R. RStudio, Inc., Boston, MA, United States) or SPSS (IBM, SPSS Inc., Armonk, NY, United States).

## Results

### Effects of sound localization training on task performance

Over the course of collecting behavioral data on the sound-localization task, we observed little change in the performance of the ferrets under normal hearing conditions (i.e., during the testing runs carried out without an earplug) (Figure 1). The accuracy of their responses on the approach-to-target task improved as the duration of the broadband target sound was increased (from 47.8 ± 3.4% correct for 40 ms to 96.1 ± 1.5% correct for 2,000 ms) (GLMM, *p* < 0.0001) (Figures 1C, D). Despite extensive testing, which included several periods of monaural occlusion (Figure 1B), their performance across different stimulus durations remained very stable when normal binaural inputs were available, other than a small reduction in percentage correct scores at intermediate target durations (100–500 ms) (GLMM, *p* = 0.001) (Figure 1C). The lack of improvement in localization accuracy over time for broadband stimuli providing access to the full range of spatial cues suggests that no perceptual learning had taken place. Furthermore, the consistent performance across different stimulus locations (Figure 1D) gave no indication of any long-lasting change in the way these cues were processed and integrated under normal hearing conditions, despite the ferrets experiencing several intervening periods during which they learned to adapt their behavior to conductive hearing loss in one ear.

### Behavioral adaptation to unilateral conductive hearing loss

**FIGURE 2:**
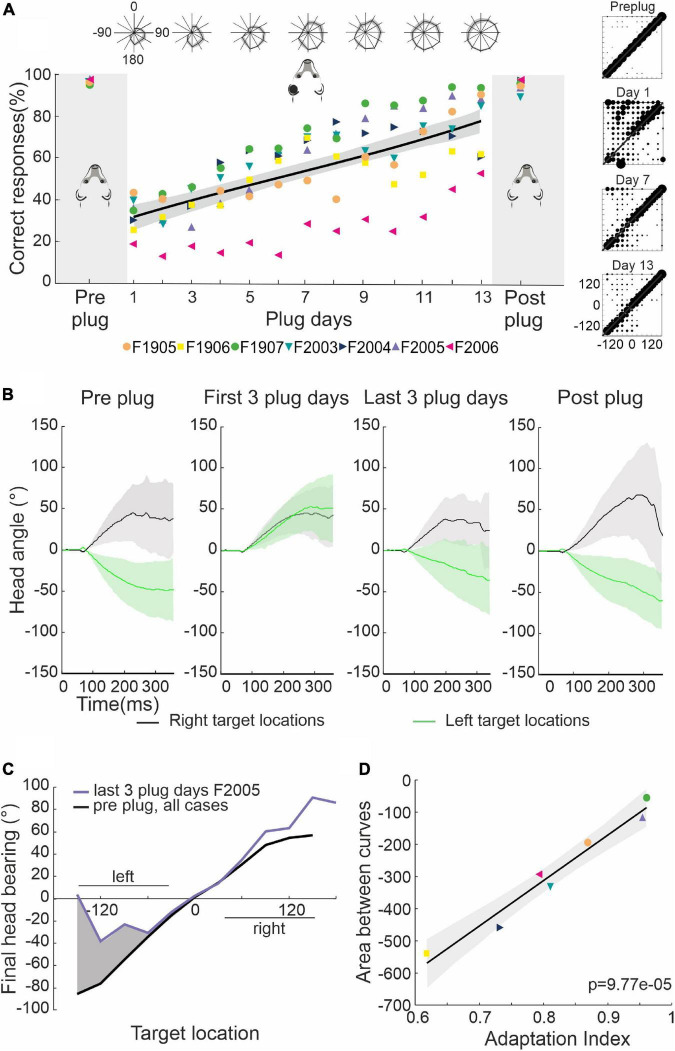
Adaptive changes in approach-to-target and head orienting responses over the course of monaural occlusion. **(A)** Percentage correct scores for the approach-to-target behavior. Different symbols indicate individual ferrets. Their mean scores over 3 days prior to earplug insertion and 3 days after earplug removal are highlighted by the gray regions on either side of the scores obtained for each day of training with the left ear occluded, which for these animals lasted 13–16 days (days 1–13 only are shown). The black line and shaded area represent the linear regression and the 95% confident intervals for the data obtained with the earplug in place. The polar plots at the top show the mean ± S.E. score across animals at different target locations for every 2 days of training. The stimulus-response plots on the right show the distribution of response locations selected (*y*-axis) as a function of target location (*x*-axis) before and at three different time points after earplug insertion. **(B)** Mean ± S.E. changes in head angle for left (green) and right (black) target locations for ferret F2005 (measured at 500 FPS and low-pass filtered at 50 Hz for plotting). Positive values correspond to rightward turns and negative values correspond to leftward turns of the head. Plots from left to right correspond to the 3 days before earplug insertion, the first 3 days with the ear plugged, the last 3 days with the ear plugged, and the 3 days following earplug removal. Occluding the left ear disrupted head orienting responses made to locations on the left, which progressively recovered as approach-to-target performance improved with training. **(C)** Mean final head bearing versus target location before earplug insertion averaged across all animals (black) and for the last 3 days of earplug adaptation for the example animal F2005 (purple). The shaded region represents the area between the two curves on the side of the occluded ear, computed as an index of adaptation of the head orienting responses. **(D)** Relationship between the adaptation index (ratio of the percentage correct scores on the last 3 days of plugging and before earplug insertion), which reflects recovery with training of the approach-to-target behavior, and adaptation of the head orienting responses (the area between the head orientation curves, as in panel **C**) for all seven animals shown in panel **(A)**.

The approach-to-target responses of one cohort of ferrets (*n* = 7) are shown in Figure 2A. They localized 1,000-ms broadband noise busts with a consistently high level of accuracy when normal binaural and monaural spatial cues were available, with their performance declining from ∼95% in the last pre-plug session to ∼30% correct when one of their ears (the left in this case) was first occluded. The ferrets initially mislocalized almost every stimulus presented in the frontal region of the hemifield ipsilateral to the earplug (see polar plots at the top of this figure). Over the first few days of monaural occlusion, a gradual improvement in performance occurred at all other locations, including those on the side of the open ear, which was then followed by a recovery in localization accuracy at the frontal ipsilateral locations. This pattern of adaptation was remarkably consistent across animals.

We also measured the accuracy of the ferrets’ sound-evoked head orienting movements, as these are thought to involve different neural circuits from those required for the approach-to-target responses (Thompson and Masterton, 1978; Lomber et al., 2001; Nodal et al., 2012; Isa et al., 2021; Figures 2B, C). Data from one example ferret (F2005) show that when the earplug was inserted, the initial head turns made following sound presentation were clearly biased toward the side of the open ear, independent of the location of the target (Figure 2B, first 3 plug days). Adaptation of the head orienting movement then occurred with training on the sound-localization task, which was manifest as a gradual recovery in orienting responses toward the side of the plugged ear for stimulus locations on that side (Figure 2B, last 3 days). It should be noted, however, that the degree of adaptation varied between animals and was generally not complete, as shown by the undershoot in the responses made in that hemifield by ferret F2005 on the last 3 plugging days (Figure 2C). This mirrors the asymmetry in the approach-to-target behavior at the corresponding stage (Figure 2A). As with the approach-to-target responses, the metrics of the head turns measured on the days after earplug removal resembled the pre-plug data (Figure 2B).

Adaptation of the head orienting responses was quantified for each ferret as the area between the average final head bearing across all animals prior to earplug insertion and the final head bearing measured for individual animals on the side of the occluded ear in the last 3 days of training (Figure 2C). This was then compared to the adaptation index derived from the approach-to-target behavior, which was computed as the ratio between the percentage correct responses in the last 3 days of monaural occlusion and the 3 days prior to earplug insertion. The comparison of these two measures for individual animals revealed that they were highly correlated (slope significantly different from 0; *R*^2^ = 0.94, *p* < 0.001) (Figure 2D), with the ferrets that achieved the highest percentage correct scores also showing greatest adaptation of the head orienting responses, as reflected by a smaller difference between pre-plug and adapted final head bearing.

### Equivalent adaptation to occlusion of each ear

**FIGURE 3:**
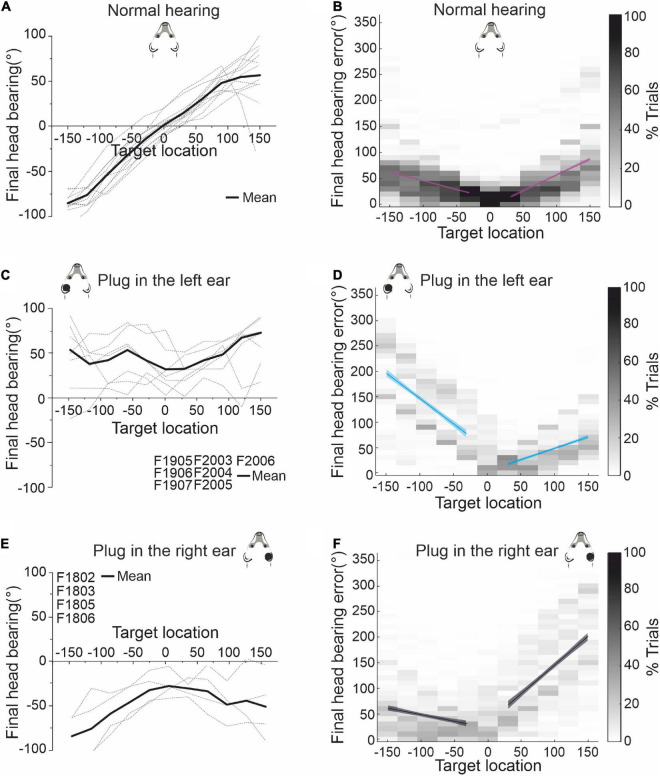
Effect of plugging either the left or the right ear on the accuracy of sound-evoked head-orienting responses. **(A)** Average final head bearing versus target location in normal hearing conditions for each of the animals (gray dashed lines) that participated in this study. Positive values correspond to targets on the right and rightward head movements, and negative values to targets on the left and leftward movements. The solid line represents the mean across animals. **(B)** Mean final head bearing errors versus target location in normal binaural hearing conditions for all animals. Linear regressions and their 95% confidence intervals are fitted independently for right and left target locations (magenta lines). **(C)** Mean final head bearing versus target location in the first 3 days with the left ear occluded. **(D)** Mean final head bearing errors versus target location in the first 3 days with the left ear occluded. Linear regressions and their 95% confidence intervals are fitted independently for right and left target locations (blue). **(E)** Mean final head bearing versus target location in the first 3 days with the right ear occluded. **(F)** Mean final head bearing errors versus target location in the first 3 days with the left ear occluded. Linear regressions and their 95% confidence intervals are fitted independently for right and left target locations (black/gray).

Before investigating the effects of further exposure to unilateral conductive hearing loss in either the same or the contralateral ear, it was first necessary to show that ferret localization behavior adapts equally well to occlusion of either the left or the right ear. This is illustrated in Figure 3 for the sound-evoked head orienting responses recorded from two groups of ferrets, one of which was plugged in the left ear (the animals used in Figure 2) and another in the right ear. In the normal hearing (pre-plug) sessions, the final head bearing of the ferrets varied systematically with the target location out to ±150° azimuth (Figure 3A). As previously reported (Nodal et al., 2008), we observed an increasing undershoot in the final head bearing for progressively more eccentric targets (Figure 3B). There was no difference in the accuracy of the responses to targets in the left and right hemifields or in the absolute slope of the regression lines fitted to these data (GLMM, *p* = 0.055; Table 1).

**TABLE 1:** Parameters of the fitted regression lines for head bearing errors in Figure 3.

	y = a + bx	*R* ^2^	a (intercept)	b (slope)	*p*_*b*_	tStat (b)
Preplug	Left side	0.17	11.96	-0.333	<0.0001	*t*_(2049)_ = −20.220
	Right side	0.28	-4.00	0.610	<0.0001	*t*_(1822)_ = 26.674
Plug in the right ear	Left side	0.05	22.69	-0.258	<0.0001	*t*_(854)_ = −7.102
	Right side	0.34	33.55	1.124	<0.0001	*t*_(833)_ = 20.829
Plug in the left ear	Left side	0.34	47.17	-1.000	<0.0001	*t*_(762)_ = −19.805
	Right side	0.26	3.86	0.449	<0.0001	*t*_(777)_ = 16.784

The head-orienting responses were affected in exactly the same way by plugging either the left ear in one group of ferrets (Figures 3C, D) or the right ear in the other group (Figures 3E, F). The final head bearing was initially biased toward the side of the open ear, i.e., to the right when the left ear was plugged (Figure 3C) and to the left when the right ear was plugged (Figure 3E). Although the size of the head orienting errors across locations remained unchanged on the side of the open ear, much larger errors were made on the contralateral side, as indicated by the steeper slope of the linear fits to these data (Figures 3D, F and Table 1). This asymmetry in performance between the left and right hemifields then declined with training, as illustrated by the adaptive changes in head-orienting behavior for one example animal (F2005) in Figure 2B.

### Adaptive learning is partially retained when spatial cues are altered again

Previous work has suggested that adaptation to the altered cues resulting from monaural occlusion leave a “memory trace,” since a second period of ear-plugging has a smaller impact on localization accuracy (Kacelnik et al., 2006; Bajo et al., 2019). Here, we explored further how this spatial learning and its memory or retrieval are affected by multiple periods of monaural occlusion and their relative timing. To that aim, the interval between the training periods was varied, as was the ear that was plugged.

**FIGURE 4:**
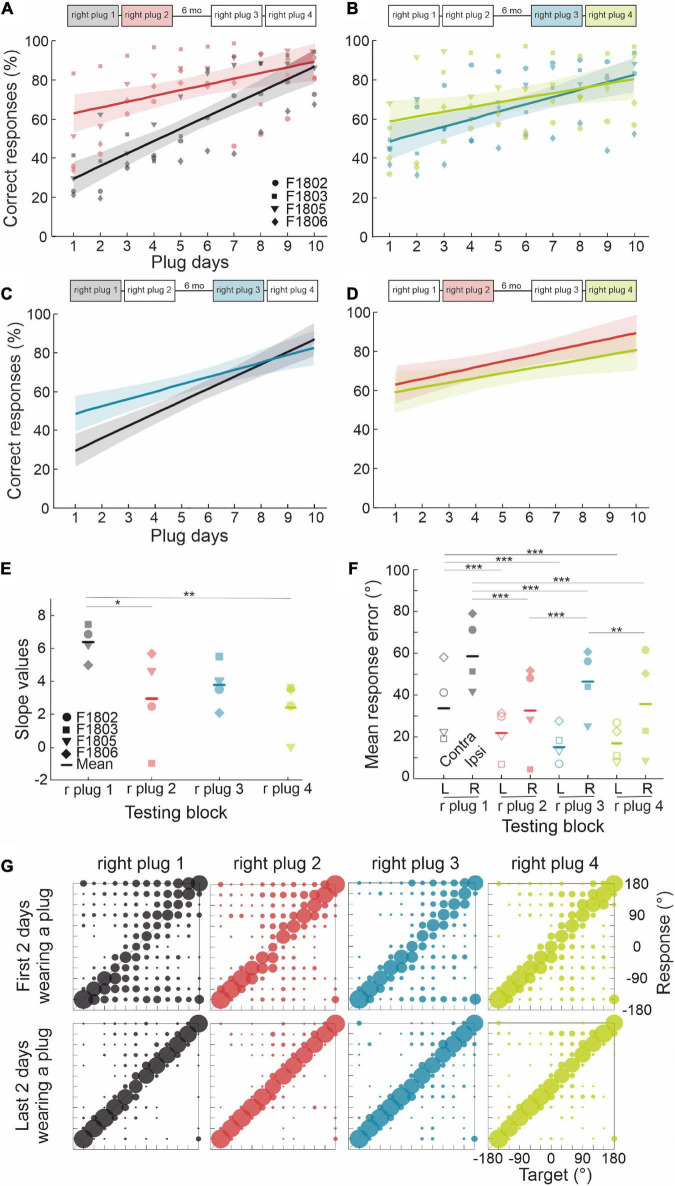
Effect of repeated occlusion of the same ear on adaptive learning. **(A)** Regression lines (shaded areas indicate 95% confidence intervals) and individual animal scores during the first (right plug 1, black line and gray symbols) and second (right plug 2, red line and symbols) periods of right ear occlusion, which were separated by 7 days of normal binaural hearing. **(B)** Regression lines and individual performance for the third (right plug 3, blue line and symbols) and fourth (right plug 4, green line and symbols) periods of right ear occlusion, which were also separated by 7 days of normal binaural hearing. A longer interval of 6 months separated the right plug 2 and right plug 3 runs. **(C)** Comparison of regression lines for the right plug 1 and right plug 3 runs, which were each preceded by long periods (∼6 months) of normal binaural hearing. **(D)** Comparison of regression lines for the right plug 2 and right plug 4 runs, which were each preceded by 7 days of normal binaural hearing following the right plug 1 and right plug 3 runs, respectively. **(E)** Individual animal and mean regression slopes for all four periods of monaural occlusion. **(F)** Mean response error magnitude in the initial 2 days of each period of right ear occlusion (right plug 1 to 4). Errors are shown separately for ipsilateral (R, right) and contralateral (L, left) target locations. Midline locations (0° and 180°) were not included in this analysis. Asterisks represent statistical significance of paired comparisons (*p < 0.05, **p < 0.01, and ***p < 0.001). **(G)** Response probabilities versus target location for the initial 2 days (top row) and the final 2 days (bottom row) of each of the four periods of right ear occlusion. For the numerical values and statistical analysis, refer to the main text.

The ferrets in this group (*n* = 4) first experienced four periods of right ear occlusion, separated by short (7 days) or long (6 months) periods of normal binaural hearing. To determine whether they were then able to adapt to a different set of altered spatial cues, we subsequently measured their performance when the contralateral left ear was occluded instead (see Figure 1B for timeline). We modeled adaptation to an earplug by fitting a simple regression line, for which the independent term (a) is inversely proportional to the initial disruption caused by insertion of the plug and the slope (b) represents the rate of adaptation (Table 2). Plugging the right ear for the first time (right plug 1) resulted in the most dramatic loss of sound localization accuracy from 97.6 ± 1.2% correct to 28.9 ± 9.3%, as indicated by the lowest value of the intercept (*a* = 10.366). Sound localization accuracy recovered with twice-daily training and the highest rate of adaptation (*b* = 6.4) was observed during this first period of monaural occlusion (Figure 4A). On all three subsequent occasions that the same ear was plugged, a smaller initial drop in performance was observed (Figure 4 and see intercepts in Table 2). Although less adaptation was therefore required, the animals’ performance improved at a slower rate (less steep slopes) than during the first adaptation period, each time reaching a mean score of 70–80% correct after 10 days of monaural occlusion. All adaptation slopes were, however, statistically significant from zero (Table 2), indicating that the ferrets were relearning to accommodate the altered spatial cues after each period of normal binaural experience.

**TABLE 2:** Parameters of the fitted regression lines during earplug adaptation.

y = a + bx	*R* ^2^	a (intercept)	b (slope)	*p*_*b*_	tStat (b)
Right plug 1	0.633	10.366	6.377	5.06e−10	*t*_(38)_ = 8.2695
Right plug 2	0.198	54.064	2.939	0.00234	*t*_(38)_ = 3.2623
Right plug 3	0.337	37.354	3.775	5.07e−05	*t*_(38)_ = 4.5681
Right plug 4	0.122	51.785	2.395	0.0154	*t*_(38)_ = 2.5363
Left plug 1	0.474	20.61	3.541	5.43e−07	*t*_(38)_ = 6.0149
Left plug 2	0.442	33.301	3.524	1.71e−06	*t*_(38)_ = 5.6525

Despite the similar pattern of adaptation in the second, third and fourth periods of right ear occlusion, differences were observed based on the interval between the plugging runs. The right plug 2 and right plug 4 runs were preceded by a short period (7 days) of normal binaural hearing following a prior period of monaural occlusion (right plug 1 and right plug 3, respectively) and had the smallest initial localization errors when the earplug was first inserted and lowest slope values (Figures 4A, B, D, E and Table 2). This, therefore, suggests a greater retention of the previously learned adaptation. By contrast, the third period of right ear occlusion (right plug 3) was preceded by 6 months of normal hearing, and the adaptation profile more closely resembled that for right plug 1, i.e., a larger initial drop in performance (lower intercept value) and faster rate of adaptation (higher slope) than in the right plug 2 and right plug 4 runs (Figures 4C, E and Table 2).

The distribution of the localization errors produced by monaural occlusion was not homogeneous across target locations and was always greater in the hemifield ipsilateral to the occluded right ear. This pattern was apparent at the start of all 4 ear-plugging runs (Figures 4F, G top row), and partly reflects mis-localization of sounds presented on the side of the earplug to the opposite side (right-to-left errors were initially twice as high as left-to-right errors). However, in contrast to the first and third periods of right ear occlusion, the difference between the mean response errors in the left and right hemifields for the right plug 2 and 4 runs was not significant (GLMM, *p* = 0.126, *p* = 0.99, respectively) (Figure 4F), providing further evidence that the amount of prior normal binaural experience determines how the brain responds behaviorally to each episode of unilateral conductive hearing loss.

The spatial pattern of errors also provides insights into the potential basis for the recovery of sound localization accuracy at different stages during the adaptation process. Localization errors on the side of the open left ear were consistently low at the start of the second, third and fourth periods of right ear occlusion (Figures 4F, G), which could reflect a rapid switch to the use of monaural cues provided by the open ear. A greater and more gradual improvement in performance was found on the side of the plugged ear (Figure 4G) and over this time the incidence of both left-right and front-back errors declined. This could be due to a more gradual remapping of the abnormal binaural cues to compensate for the effects of the earplug. Further support for this interpretation is provided by the observation that performance reaches ≥70% correct after a similar number of days irrespective of previous earplug experience and adaptation, the time elapsed between the periods of monaural occlusion, or the initial impact of plugging the ear on localization performance.

We have previously shown that the primary auditory cortex (A1) plays an essential role in adapting to an earplug in one ear, but is not required for retrieval of the memory trace that facilitates adaptation during a second period of monaural occlusion (Bajo et al., 2019). This raises the possibility that adaptive learning is consolidated in circuits that lie downstream from the cortex. The disruptive effects of auditory corticocollicular lesions on adaptation (Bajo et al., 2010) suggest that midbrain areas that are known to be more directly involved in the control of head orienting behavior may be important for the retrieval of learning. We therefore also examined the impact of a second period of monaural occlusion on the accuracy of the sound-evoked head movements.

**FIGURE 5:**
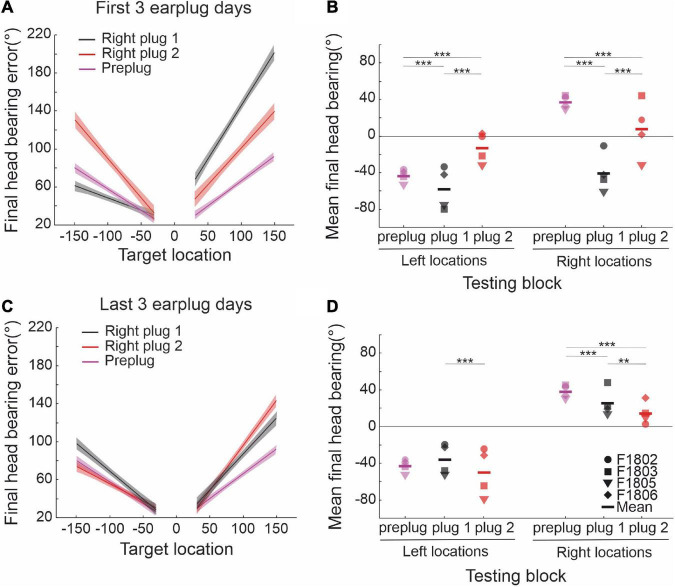
Persistence of adaptation in the sound-evoked head orienting responses. **(A)** Linear regression fits and their 95% confidence intervals for the mean final head bearing errors in the first 3 days of the right plug 1 and 2 runs (black and red lines, respectively). For comparison linear fits for preplug responses are shown in magenta. **(B)** Mean final head bearing shown separately for left and right target positions in the first 3 days of the right plug 1 and 2 runs. Midline locations (0° and 180°) were excluded. Note that the head orienting responses were no longer as biased toward the side of the open left ear at the start of the second period of right ear occlusion. **(C)** Linear regression fits and their 95% confidence intervals for the mean final head bearing errors in the pre-plug responses (magenta lines) and in the last 3 days of the right plug 1 and 2 runs (black and red lines, respectively). **(D)** Mean final head bearing shown separately for left and right target positions in the last 3 days of the right plug 1 and 2 runs. (**p < 0.01, ***p < 0.001).

In contrast to the start of the first period of right ear occlusion (right plug 1), where a marked left-right asymmetry was observed in the size of the head orienting errors due to the animals consistently turning toward the side of the open ear, these responses were more symmetrically distributed to appropriate sides of the midline at the start of the second period of right ear occlusion (right plug 2) (Figures 5A, B and Table 3). In both cases, head orienting accuracy had partially recovered toward pre-plug values by the last 3 days of ear-plugging, with all the animals now responding to the appropriate side of space (Figures 5C, D). This pattern of adaptation in head orienting behavior across the two periods of right ear occlusion is consistent with that seen for the approach-to-target responses in showing that previous experience with altered spatial cues enables the ferrets to more readily adapt to a second period of unilateral conductive hearing loss in the same ear. The head orienting data therefore support the possibility that retrieval of learning following a short period of normal hearing involves subcortical circuits.

**TABLE 3:** Parameters of the fitted regression lines for head bearing errors in Figure 5.

		y = a + bx	*R* ^2^	a (intercept)	b (slope)	*p*_*b*_	tStat (b)
	Preplug	Left side	0.14	12.84	-0.449	<0.0001	*t*_(992)_ = −12.946
		Right side	0.21	14.34	0.520	<0.0001	*t*_(977)_ = 16.388
First 3 days	Right plug 1	Left side	0.05	22.69	-0.258	<0.0001	*t*_(854)_ = −7.102
		Right side	0.34	33.55	1.124	<0.0001	*t*_(833)_ = 20.829
	Right plug 2	Left side	0.27	7.30	-0.824	<0.0001	*t*_(494)_ = −13.763
		Right side	0.24	23.19	0.779	<0.0001	*t*_(529)_ = 13.058
Last 3 days	Right plug 1	Left side	0.22	10.38	-0.587	<0.0001	*t*_(716)_ = −14.299
		Right side	0.27	12.00	0.752	<0.0001	*t*_(641)_ = 15.414
	Right plug 2	Left side	0.12	19.15	-0.364	<0.0001	*t*_(849)_ = −10.885
		Right side	0.35	1.21	0.950	<0.0001	*t*_(837)_ = 21.099

### Adaptation to unilateral conductive hearing loss in the contralateral ear

**FIGURE 6:**
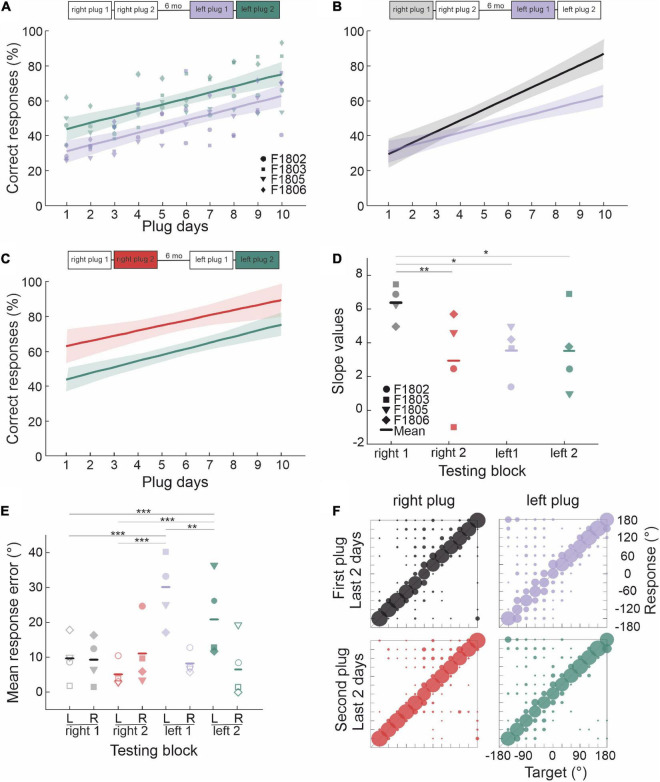
Effect of occluding the other ear following adaptation to unilateral conductive hearing loss. **(A)** Regression lines (shaded areas indicate 95% confidence intervals) and individual animal scores during the first (left plug 1, purple line and symbols) and second (left plug 2, green line and symbols) periods of left ear occlusion, which were separated by 7 days of normal binaural hearing. Unlike the right plug 1 and right plug 2 runs (Figure 4A) previously experienced by these animals, the slopes of the linear fits to these data were very similar. **(B)** Comparison of the regression lines for the right plug 1 (black/gray) and left plug 1 (purple) runs. Although the ferrets performed at the same level when each ear was first plugged, much less adaptation was seen when the hearing loss was experienced in the left ear following adaptation to right ear occlusion. **(C)** Comparison of the regression lines for the right plug 2 (red) and left plug 2 (green) runs. The slopes are very similar, but the difference in intercepts indicates less overall improvement in localization accuracy when the left ear was occluded. **(D)** Individual animal and mean regression slopes for the fits shown in panels **(A–C)**. **(E)** Mean response error magnitude in the final 2 days of each of these periods of monaural occlusion (right plug 1 and 2, left plug 1 and 2). Errors are shown separately for target locations ipsilateral (filled symbols) and contralateral (open symbols) to the side of the earplug. Midline locations (0° and 180°) were not included in this analysis. Asterisks represent statistical significance of paired comparisons (*p < 0.05, **p < 0.01, and ***p < 0.001). **(F)** Response probabilities versus target location for the final 2 days of the first right and left (top row) and the second right and left (bottom row) periods of monaural occlusion.

We next examined whether ferrets that had learned to localize accurately following occlusion of one ear also exhibit similar adaptive plasticity when the direction of the binaural imbalance is reversed by plugging the other ear. The same group of ferrets that had experienced four periods of right ear occlusion now had their left ear plugged (Figure 6). This resulted in an initial drop in localization accuracy to ∼30% correct, the same as that seen when the right ear was plugged for the first time (Figures 6A, B).

All the ferrets then exhibited a gradual improvement in performance with training, but they did so at a slower rate than in the equivalent first right ear plug run and recovered to a much lower level after 10 days of training with the left earplug in place (Figures 6B, D and Table 2). A further difference in localization accuracy between right plug 1 and left plug 1 was revealed by comparing the responses to sounds presented in the left and right hemifields. In the first left earplug run, localization accuracy at the end of the adaptation period was significantly worse on the side of the plugged ear than on the side of the open ear (repeated measures ANOVA: effect of side, *p* = 0.01), whereas no difference between the left and right hemifields was found for the first right earplug run (*p* = 0.139) (Figures 6E, F). This is not because of any inherent difference in the capacity of the auditory system to adapt to conductive hearing loss in the two ears (Figure 3), nor is it likely to reflect any residual bias toward the side of the previously open ear since this declined as the ferrets adapted and disappeared when the earplug was removed. Rather, it appears to reflect the prior experience of the ferrets in repeatedly adapting to occlusion of the right ear.

A week after removing the earplug from the left ear, this ear was occluded for a second time (left plug 2). As with repeated plugging of the right ear, this resulted in a much smaller initial deficit than when the animals first received an earplug in the left ear (Figure 6A and Table 2), and their performance then improved with training at approximately the same rate as in left plug 1 (Figures 6A, D) and right plug 2 (Figures 6C, D). However, the animals localized less accurately at the end of the second period of left ear occlusion than after equivalent training with the right ear plugged (Figure 6C), due to their poorer performance on the left side, ipsilateral to the earplug, relative to the right side (repeated measures ANOVA, *p* = 0.003) (Figure 6E). These results therefore show that learning to localize sound with one ear occluded interferes with the capacity to subsequently adapt to conductive hearing loss in the other ear and that this effect persists over more than one period of monaural occlusion.

### Adaptation to unilateral conductive hearing loss generalizes beyond the training stimulus

**FIGURE 7:**
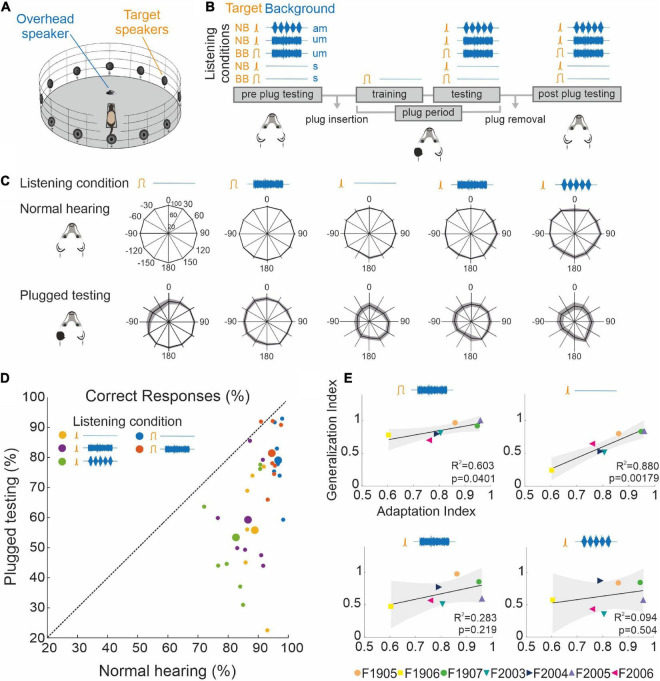
Generalization of training-dependent adaptation. **(A)** Schematic of the circular testing arena indicating the position of the peripheral target loudspeakers (as in Figure 1) and the loudspeaker located above the central platform used to generate background noise. **(B)** Timeline indicating the different combinations of target and background sounds used for behavioral training and testing. The two types of target sounds [broadband (BB) and narrowband (NB) noise] are depicted in orange and three possible backgrounds [silence (s), unmodulated BB noise (um), and BB noise amplitude modulated at 5 Hz (am)] are shown in blue. **(C)** Spatial distribution of mean (±S.E.) percent correct scores for each target and background combination under normal listening conditions (top row) and after adaptation to an earplug in the left ear (bottom row). **(D)** Mean percent correct scores for all target and background combinations under normal hearing conditions and after adaptation with one ear plugged. Large circles are the mean values and the small circles represent individual animals. Almost all values fall below the diagonal identity line, indicating more accurate responses with normal binaural inputs. Responses to BB targets were closer to the identity line and therefore showed less difference between the two hearing conditions. **(E)** Relationship between the adaptation index (the ratio of the percent correct scores for BB noise in silence in the final 3 days of adaptation to monaural occlusion and the preplug scores) and the generalization index [the ratio of the percent correct scores for each of the other stimulus types (shown at the top of the panels) measured with the earplug in place at the end of the adaptation run and the corresponding preplug scores].

To explore the extent to which the improvement in localization accuracy following adaptation to unilateral conductive hearing loss generalized beyond the standard broadband noise training paradigm, we tested the performance of a group of ferrets using a combination of different target sounds and acoustic backgrounds before and after they had been trained to adapt to an earplug. The target sounds were either broadband noise (BB), for which all acoustic spatial cues would have been available, or narrowband noise (NB) with a one-octave bandwidth centered on 16 kHz, selected to reduce the availability of binaural cues and to provide limited access to high-frequency spectral localization cues (Carlile, 1990; Keating et al., 2013). The target sounds were either presented in silence (s) or against a background of continuous broadband noise presented from an overhead speaker (Figure 7A), whose amplitude was either unmodulated (um) or modulated at 5 Hz (am) to add temporal structure to it (Figure 7B).

After measuring their localization accuracy with all combinations of targets and acoustic backgrounds (Figure 7C, Normal hearing), the animals were plugged in the left ear and trained as usual using 1,000-ms BB targets. Once they reached the adaptation criterion, the animals were retested on the above combination of target and acoustic backgrounds while still wearing the earplug (Figure 7C, Plugged testing).

Under normal hearing conditions and in a silent acoustic background, the ferrets localized the BB targets almost perfectly (96.4 ± 0.76%) and the NB target sounds slightly less well (88.9 ± 1.13%) (GLMM, *p* < 0.001, Table 4), particularly for posterior sound locations (Figure 7C, Normal hearing, Table 5). Reducing the bandwidth of the stimulus led to an increase in the incidence of both front-back errors (sounds presented in the frontal hemifield that were mislocalized into the posterior ipsilateral hemifield or vice versa; BB target, 0.94 ± 0.83%; NB target 1.99 ± 1.30%; paired sample *t*-test, df = 6, *p* = 0.039) and left-right errors (BB target, 0.23 ± 0.23%; NB target 0.81 ± 0.51%; paired sample *t*-test, df = 6, *p* = 0.043). Sound localization accuracy was also degraded to a small but significant extent in the presence of both types of background noise (GLMM, *p* = 0.019, Table 4).

**TABLE 4:** Generalized linear mixed model comparing different stimuli.

			Normal hearing					After adaptation				
			BBs	BBum	NBs	NBum	NBam	BBs	BBum	NBs	NBum	NBam
Reference	Normal hearing	BBs	−	−0.171	−0.610	−0.728	-0.915	-1.080	-0.924	-1.740	-1.613	-1.799
		BBum	**	−	−0.439	−0.556	-0.744	-0.909	-0.753	-1.569	-1.441	-1.628
		NBs	***	***	−	−0.117	-0.305	-0.470	-0.314	-1.130	-1.003	-1.189
		NBum	***	***	**	−	-0.188	-0.352	-0.196	-1.012	-0.885	-1.071
		NBam	***	***	***	***	−	-0.165	-0.009	-0.825	-0.698	-0.884
	After adaptation	BBs	***	***	***	***	***	−	0.156	-0.660	-0.533	-0.719
		BBum	***	***	***	***	0.816	***	−	-0.816	-0.689	-0.875
		NBs	***	***	***	***	***	***	***	−	0.127	-0.059
		NBum	***	***	***	***	***	***	***	***	−	-0.186
		NBam	***	***	***	***	***	***	***	0.081	***	−

Target: BB, broadband noise; NB, narrowband noise (one octave bandwidth, centered on 16 kHz). Background: s, silence; um, unmodulated noise; am, amplitude-modulated noise. **p < 0.01 and ***p < 0.001; numbers are odds ratios.

**TABLE 5:** Sound localization accuracy for different stimuli.

% Correct responses					
Normal hearing/After adaptation					
Ferret#	BBs	BBum	NBs	NBum	NBam
F1905	96.2/82.9	94.8/92.0	86.3/68.9	87.2/85.4	90.6/76.1
F1906	98.2/59.2	95.2/74.4	93.0/22.5	91.6/44.0	76.6/44.0
F1907	97.9/92.8	97.5/90.7	91.8/76.8	91.5/79.1	90.6/77.5
F2003	96.4/77.7	94.6/78.0	91.0/47.5	90.9/47.5	84.9/31.0
F2004	95.2/75.3	95.5/77.2	85.8/45.0	76.5/59.8	72.0/63.6
F2005	95.4/91.6	91.0/91.8	88.0/73.9	83.0/49.8	79.5/44.6
F2006	97.1/73.9	93.1/65.9	86.3/56.0	85.6/49.3	84.0/37.0

Target: BB, broadband noise; NB, narrowband noise (one octave bandwidth, centered on 16 kHz). Background: s, silence; um, unmodulated noise; am, amplitude-modulated noise.

Following training with one ear plugged, the ferrets showed the usual substantial recovery in their ability to localize BB noise bursts. Nevertheless, their sound localization accuracy across all stimuli was less accurate than that measured under normal hearing conditions prior to plugging one ear (Figures 7C, D and Table 4, GLMM, hearing condition, *p* < 0.001). This was particularly the case for the NB targets, with a greater difference in performance between BB and NB targets when one ear was occluded than prior to ear-plugging (GLMM, *p* < 0.001), as indicated by the greater distance to the identity line for the data from the NB targets in Figure 7D. The difference in localization performance with target bandwidth was also apparent from the incidence of front-back errors (BB target, 3.82 ± 2.92%; NB target 10.42 ± 6.22%; paired sample *t*-test, df = 6, *p* = 0.002) and left-right errors (BB target, 2.78 ± 2.43%; NB target 8.27 ± 6.19%; paired sample *t*-test, df = 6, *p* = 0.011). Following adaptation to monaural occlusion, the ferrets localized NB targets significantly worse on the side of the plugged ear (GLMM effect of target location, *p* < 0.001; odds ratio −0.385 relative to the best localized locations) (Figure 7C, Plugged testing).

Surprisingly, we found an improvement in performance in the presence of background noise for BB target sounds after the animals had adapted to the earplug (GLMM, *p* < 0.001, odds ratio = 0.156) (Figures 7C, D). This is also reflected by the greater value of the generalization index, the mean performance following adaptation relative to that obtained under normal hearing conditions (Figure 7E and Table 6). We observed a significant correlation between the degree of adaptation and generalization: ferrets that showed more complete recovery in their performance with training also localized BB targets against background noise more accurately, indicating that they were able to effectively segregate these sound sources despite the presence of a conductive hearing loss in one ear.

**TABLE 6:** Adaptation and generalization indices.

	Adaptation index	Generalization index			
Ferret#	BBs	BBum	NBs	NBum	NBam
F1905	0.86	0.97	0.80	0.98	0.84
F1906	0.60	0.78	0.24	0.48	0.57
F1907	0.95	0.93	0.84	0.86	0.85
F2003	0.80	0.82	0.52	0.52	0.36
F2004	0.79	0.81	0.52	0.78	0.88
F2005	0.96	1.01	0.84	0.60	0.56
F2006	0.76	0.71	0.65	0.57	0.44
Mean ± SD	0.82 ± 0.12	0.86 ± 0.11	0.63 ± 0.22	0.69 ± 0.19	0.64 ± 0.21

Greater inter-animal variability in performance was found with NB than BB target sounds (Figures 7C–E and Table 6). Ferrets that adapted more to the earplug when trained with BB sounds also localized NB targets in silence more accurately. Although they localized less accurately than with BB noise (Figures 7C, D), the significant correlation between the adaptation index and the generalization index (Figure 7E) suggests that sufficient spatial information that formed the basis for adapting to unilateral conductive hearing loss remained in these NB stimuli. The addition of background noise weakened this relationship, particularly when it was amplitude modulated, suggesting that background noise has a more disruptive effect on the transfer of learning when more limited spatial cues are provided by the target stimuli.

### Role of spectral cues and dynamic cues in adaptation to unilateral conductive hearing loss

The lack of an aftereffect following earplug removal is consistent with a primary adaptation strategy to unilateral conductive hearing loss in which the animals learn to rely more on the intact monaural spectral cues provided by the non-occluded ear than the normally dominant but now distorted binaural cues. However, adaptive changes in binaural cue sensitivity appear to contribute as well, as shown by the recovery in localization accuracy observed in humans when pure tones are interspersed during training with one ear occluded (Keating et al., 2016). Our finding that adaptation of sound localization in the horizontal plane is initially restricted to the side of the open ear, with improvements in performance on the plugged side occurring later during the period of monaural occlusion also supports the possibility that more than one process may underlie this training-dependent plasticity.

An additional factor that needs to be considered is that the relatively long training stimulus (1,000 ms noise bursts) may have allowed the animals to sample dynamic spatial cues as they were approaching the perceived sound source location. The very similar pattern of adaptation observed in the head orienting responses (Figure 2), which have a latency of ∼100–200 ms (Nodal et al., 2008, 2012), strongly suggests that adaptation relies primarily on the spatial information available at the onset of the target sound. Nevertheless, the extent of the adaptation has been shown to increase with the stimulus duration (Kacelnik et al., 2006), which could reflect greater integration time or multiple sampling of the available localization cues.

To gain a better understanding of the processes underlying training-dependent adaptation to unilateral conductive hearing loss, we interspersed sessions in which high-frequency narrowband sounds [1/6 octave bandwidth centered on 15 kHz, as in Keating et al. (2015)] were presented during the adaptation training with broadband noise. These sounds were designed to eliminate monaural spectral cues and ITDs, leaving only narrowband ILDs available. In addition, the stimuli in these trials were either 200 ms or 1,000 ms in duration to determine whether more adaptation occurred with the longer duration sounds.

**FIGURE 8:**
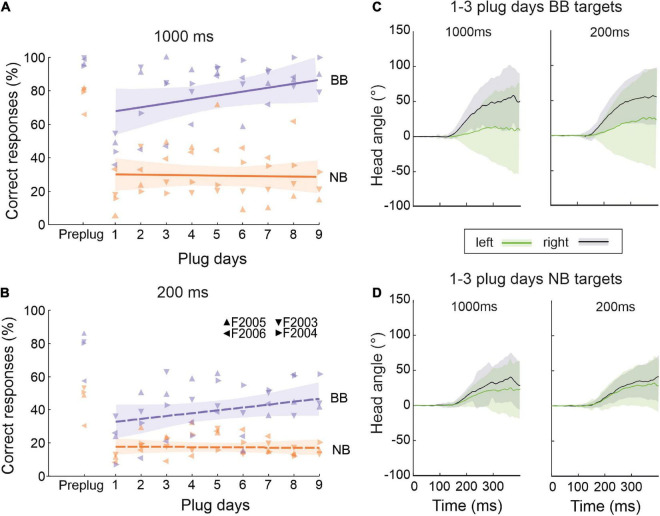
Role of dynamic and spectral cues in adaptation to unilateral conductive hearing loss. **(A)** Percentage of correct responses for 1,000-ms broadband noise (purple, BB) and 1/6 octave narrowband noise bursts with a center frequency of 15 kHz (orange, NB) over the course of daily training with one ear occluded. Colored lines are linear regressions, and the shaded areas correspond to the 95% confidence intervals. The symbols represent individual animals. **(B)** Same as panel **(A)** but for stimuli with a duration of 200 ms. **(C)** Change in the mean (±S.E.) horizontal angle of the head over time following BB target onset at time 0 ms. These measurements were taken at the start (days 1–3) of the ear-plugging run, combined across target locations in the left *(green)* and right *(black)* hemifields, and are plotted separately for 1000-ms (left) and 200-ms (right) noise bursts. **(D)** Same as panel **(C)** but for NB target sounds.

As expected, the pre-plug scores showed that the ferrets localized high-frequency narrowband targets less accurately than the broadband sounds at stimulus durations of both 1,000 ms (Figure 8A) and 200 ms (Figure 8B). Including different stimulus types and durations within the training block did not interfere with the expected adaptation to BB noise stimuli, with the percentage correct scores increasing throughout the period of monaural occlusion for both target durations (GLMM testing day, *p* < 0.001) (Figures 8A, B). By contrast, no improvement in performance was seen over time for NB targets that had a duration of either 1,000 ms (GLMM, testing day, *p* = 0.071) (Figure 8A) or 200 ms (GLMM, testing day, *p* = 0.341) (Figure 8B).

The sound-evoked head movements measured for these animals (combined across target locations in each hemifield for the first 3 days of monaural occlusion and plotted separately for BB and NB stimuli in Figures 8C, D, respectively) were very similar at each of the two stimulus durations used. These averaged measurements demonstrate that the head began to move near the end of the 200-ms noise bursts, and this movement continued during the longer stimulus until the animal left the central start platform [which typically occurs at ∼600 ms after stimulus onset (Kacelnik et al., 2006; Nodal et al., 2008)]. Although the metrics of the head turns were little affected by stimulus duration, they differed with the bandwidth of the stimulus in a similar way to the approach-to-target response accuracy shown in Figures 8A, B.

These results therefore indicate that that 1/6-octave bandwidth noise bursts centered at 15 kHz do not contain sufficient spatial information to support the adaptive improvements in localization performance that take place during the training runs with BB noise. Furthermore, localization accuracy was reduced for both BB (GLMM, target duration, *p* < 0.001) and NB (GLMM, target duration, *p* < 0.001) stimuli when the duration used was too short for the animals to be able to use dynamic cues to any degree, suggesting that the ferrets may benefit from re-sampling the longer stimuli during and after the initial head turn. Nevertheless, some adaptation still took place with 200-ms noise bursts so long as they were sufficiently broadband to provide access to other auditory spatial cues (Figures 8A, B). Adaptive learning does not therefore depend on head movements, though it is possible that they enhance this process.

## Discussion

Perceptual learning enables auditory perceptual skills to be improved with practice or training (Irvine, 2018), and allows abnormal inputs to be accommodated in ways that are likely to be important in the treatment of hearing disorders (Kumpik and King, 2019; Glennon et al., 2020). Plasticity in the processing of auditory spatial information is essential for calibrating neural circuits through experience with the available cues, which vary with individual differences in the geometry of the head and ears and change over time, particularly as these structures grow but potentially also following hearing loss. While this plasticity is most pronounced during development, many studies have shown that the adult brain can learn to utilize abnormal auditory space cues (Carlile, 2014; Mendonça, 2014). We used a well-established paradigm for investigating the adaptive capabilities of the auditory system by temporarily plugging one ear in order to induce a reversible binaural imbalance, so that we could explore the retention and retrieval of learning and its generalization beyond the standard stimuli conditions used for training.

The earplugs we used disrupt binaural localization cues by attenuating and delaying the sound at the occluded ear (Moore et al., 1989; Keating et al., 2013, 2016). The attenuation is frequency dependent and will therefore also distort the spectral cues available at the occluded ear, leaving the spectral filtering provided by the open ear as the only unchanged acoustic cue. Following an immediate decline in localization accuracy in the horizontal plane, we found that performance steadily improved with daily training toward the pre-plug scores, as shown in previous studies in ferrets (Kacelnik et al., 2006; Bajo et al., 2010, 2019; Nodal et al., 2010, 2012) and humans (Kumpik et al., 2010; Keating et al., 2016). Although an improvement in localization accuracy in monaurally plugged human subjects has been observed over the course of a single training session when the sound level was fixed and visually guided feedback provided (Zonooz and Van Opstal, 2019), other work in humans has shown that training has to be spread out over several days rather than compressed into a single block on 1 day in order for adaptation to occur (Kumpik et al., 2010). While this suggests that learning to localize sound after disrupting the cues in this fashion requires a period of consolidation between training sessions, daily training in monaurally occluded ferrets has been shown to produce faster and more complete adaptation than in animals that were trained every sixth day and which therefore experienced altered spatial cues over a longer period (Kacelnik et al., 2006). Related to this is the finding that human participants who have one ear plugged only during sound-localization training sessions that took place approximately every 3 days still show a gradual improvement in performance from one session to the next despite receiving normal binaural inputs in the intervening periods (Keating et al., 2016). These findings highlight the key role of training in the rapid adaptation to altered spatial cues, confirming that it represents a form of perceptual learning.

Compensatory changes in binaural cue sensitivity have been reported in adult humans who adapt to the presence of an earplug in one ear (Keating et al., 2016; Zonooz and Van Opstal, 2019) and have also been observed at a neurophysiological level in animals following several weeks of monaural occlusion during development (Keating et al., 2015) or in adulthood (Thornton et al., 2021). Nevertheless, the absence of an after-effect in the localization responses following earplug removal suggests that the auditory system primarily adapts by giving greater weight to the unchanged monaural spectral cues provided by the open ear. This conclusion is supported by the immediate reduction in percentage correct scores produced by inserting a mold in order to reshape the external ear contralateral to the earplug (Kacelnik et al., 2006) and by the greatly reduced adaptation observed when spectral cues were disrupted during training by randomizing the spectrum of the sounds (Keating et al., 2016). Monaural spectral cues also appear to contribute to the ability to localize sounds in the horizontal plane of at least some humans who are deaf in one ear (Slattery and Middlebrooks, 1994; Van Wanrooij and Van Opstal, 2004; Agterberg et al., 2014). Our results provide additional evidence for the importance of spectral cues in adaptation to unilateral hearing loss. While the ferrets exhibited an improvement in accuracy when trained to localize broadband noise stimuli, which provide access to the full range of acoustic spatial cues, no improvements were seen with narrowband sounds (1/6-octave bandwidth noise bursts centered at 15 kHz) presented during the training to restrict access to high-frequency interaural level differences.

Following earplug removal, the auditory system switches back to relying more on binaural cues for azimuthal localization. This is shown by our finding that subsequently replugging the same ear again degrades localization accuracy (see also Kacelnik et al., 2006; Bajo et al., 2019). A capacity to alternate between different sets of cues has also been demonstrated in adult humans who experience altered spectral cues by wearing ear molds for several weeks (Hofman et al., 1998; Van Wanrooij and Van Opstal, 2005; Carlile and Blackman, 2014; Trapeau et al., 2016; Watson et al., 2017). These manipulations impaired elevation responses, which then recovered as the participants learned to localize with their new ears, but when the molds were removed, restoring normal spectral cues, they were immediately able to localize as accurately as they did prior to insertion of the molds. Furthermore, upon reinsertion of the molds approximately 1 week later, localization performance was found to be no different from that measured at the end of the previous accommodation period (Carlile and Blackman, 2014). This suggests that after learning to localize with the molds in place, humans are able to interchangeably use their original own-ear spectral cues and the abnormal spectral cues they have adapted to. Brief periods of training with inappropriate spectral cues provided by using virtual auditory displays with non-individualized head-related transfer functions also result in improvements in localization performance that have been shown to persist for at least several weeks (Zahorik et al., 2006; Mendonça et al., 2013).

The effects of occluding one ear are different, however, with our results showing that additional training is required to achieve the same level of adapted performance whenever the same ear was re-plugged, with the magnitude of the initial drop in performance positively correlated with the duration of prior normal experience and greatest at the start of the first ear-plugging run. Thus, although it takes longer for the brain to adjust to the altered spatial cues resulting from monaural occlusion than to revert to normal inputs, previous ear-plugging experience leaves a “memory trace” so that less adaptation is required when the cues are changed again in the same manner. This was particularly the case for locations ipsilateral to the open ear, suggesting that upweighting of monaural spectral cues is facilitated by prior experience.

Switching the unilateral conductive hearing loss to the contralateral ear also resulted in an impairment in localization accuracy that partially recovered with training. However, the localization performance of the ferrets improved to a significantly lesser extent than when the hearing loss was previously experienced on the other side. This is consistent with evidence that ferrets raised with one ear occluded adapt by changing the relative weighting of the spectral cues in opposite directions for each ear (Keating et al., 2013). If this also happens when the animals adapt to a unilateral conductive hearing loss in adulthood, these opposing changes in cue weighting would then need to be reversed when the earplug is switched to the other ear. Alternatively, any remapping of binaural cues that accompanies adaptation to monaural conductive hearing loss, and which may account for the more gradual improvements observed at frontal regions ipsilateral to the occluded ear, is also likely to hinder the capacity of the brain to recover localization accuracy when the side of the hearing loss is changed.

In these experiments, we measured the accuracy of the ferrets’ head orienting responses following sound presentation as well as the target location they approached to receive a reward. Our previous work has shown that restricted lesions (Nodal et al., 2010) or reversible deactivation (Nodal et al., 2012) of the auditory cortex impair approach-to-target behavior, whilst preserving the accuracy of the initial sound-evoked head orienting responses, which are likely to be more dependent on midbrain circuits (Lomber et al., 2001; Isa et al., 2021). Nevertheless, the initial head turn and subsequent selection of target location are both components of the animals’ behavioral response to sounds presented from different directions and are dependent on the same spatial cues. Indeed, we have previously reported that when ferrets with normal hearing approach an incorrect target location, the preceding head turn is more closely correlated with that response than with the target location (Nodal et al., 2008).

We observed a very similar pattern of training-dependent plasticity for each measure of sound localization, including the partial retention of learning when abnormal spatial cues are again experienced by replugging the same ear. Since silencing the primary auditory cortex impairs adaptation but not the retrieval of previous learning (Bajo et al., 2019), it is likely that the latter involves downstream circuits. Although we cannot rule out the involvement of other brain areas, our previous demonstration that the descending auditory corticocollicular pathway is essential for adaptive plasticity of both head-orienting and approach-to-target behavior (Bajo et al., 2010) suggests that the midbrain may be involved in the retention of learning. Future recording studies will be required to identify exactly where and how spatial information is stored to allow the partial retrieval of localization accuracy from one period of monaural occlusion to the next. It is interesting to note, however, that these findings are consistent with the reverse hierarchy theory of perceptual learning (Ahissar and Hochstein, 2004), whereby learning proceeds in a top-down fashion from higher to lower levels of processing, where the relevant stimulus features are represented more precisely.

Adaptation of the head-orienting responses also allowed us to address the potential contribution of sensorimotor behavior to auditory localization plasticity (Zahorik et al., 2006; Aytekin et al., 2008; Parseihian and Katz, 2012; Carlile et al., 2014). Head turning accuracy is likely to depend on the spatial cues available at the onset of the target sound and is not affected by its duration (Nodal et al., 2012). Our finding that adaptive changes in head-orienting responses during initial and subsequent periods of monaural occlusion mirrored those seen for the approach-to-target behavior suggests that head movements are not necessary for training-dependent plasticity to occur. This is supported by our finding that approach-to-target accuracy showed some recovery with one ear occluded when the duration of the broadband noise bursts was reduced to 200 ms, which is close to the head-orienting latency. Partial adaptation to unilateral conductive hearing loss has also been observed in ferrets for 40-ms broadband noise bursts (Kacelnik et al., 2006), and human participants wearing a plug in one ear can re-learn to correctly identify the target loudspeaker location without first turning toward it (Kumpik et al., 2010).

Although these findings indicate that head movements do not play an essential role in adjusting auditory localization to compensate for a conductive hearing loss in one ear, the recovery in accuracy is more complete when longer duration noise bursts are used for training (see also Kacelnik et al., 2006), suggesting that movement of the head may benefit adaptation by allowing multiple sampling of the stimulus. No improvement was observed, however, when 1/6-octave narrowband noise bursts of either 200 or 1,000 ms duration were presented during the training, suggesting that even when head movements during sound presentation were possible, these stimuli provide insufficient spatial information to provide a confident assessment of target location and therefore support learning. While this highlights the importance for adaptation to unilateral conductive hearing loss of using a broadband stimulus so that the full range of localization cues is available, it seems likely that further training with narrowband sounds would eventually lead to a remapping of high-frequency ILDs (Keating et al., 2015, 2016).

Investigating the generalization of perceptual learning to untrained stimulus properties can provide valuable insights into the neural substrates of learning and retrieval (Wright and Zhang, 2009; Dosher et al., 2013). In terms of adaptation to unilateral conductive hearing loss, generalization is clearly important if this form of training-dependent plasticity is to be relevant clinically to users of cochlear implants and hearing aids. In this respect, previous studies of adaptation to abnormal spatial cues in humans are promising in demonstrating that improvements in performance do transfer to untrained sound types (Mendonça et al., 2013; Watson et al., 2017) and locations (Zahorik et al., 2006; Mendonça et al., 2013; Watson et al., 2017; Zonooz and Van Opstal, 2019), and to reverberant conditions (Watson et al., 2017). Zonooz and Van Opstal (2019) reported that while adaptation to monaural ear-plugging leads to a generalized increase in azimuth accuracy, this was accompanied by degraded elevation responses, though individuals with single-sided deafness can benefit from monaural spectral cues for localization in both dimensions (Van Wanrooij and Van Opstal, 2004).

Our results show that the adaptation achieved after training ferrets to localize broadband noise with one ear occluded was positively correlated with their ability to generalize this performance improvement to untrained octave band noise. These narrowband stimuli would have provided high-frequency binaural cues and a subset of the directional spectral features produced by the filter properties of the head and ears (Keating et al., 2013). While these cues were sufficient to support the generalization of adaptation when the narrowband target sounds were presented in silence, the addition of background noise degraded the performance of the ferrets both before and after learning. However, when broadband target sounds were used, the spatial learning resulting from training ferrets with one ear plugged in quiet conditions was preserved when the animals were subsequently tested in a noisy environment. Indeed, following adaptation to monaural occlusion, they achieved significantly higher scores when background noise was added than in a silent background, raising the possibility that localization training may enhance the perception of targets in noise when hearing is perturbed. This indicates that the benefits of daily training for hearing-impaired individuals may translate to listening conditions that more closely resemble those of real-world environments.

## Data availability statement

The raw data supporting the conclusions of this article will be made available by the authors, without undue reservation.

## Ethics statement

The animal study was reviewed and approved by the Committee on Animal Care and Ethical Review, University of Oxford.

## Author contributions

AK and FN conceived and designed the study, with contributions from AS and VB. AS, FN, and VB acquired the data. AS led the data analysis, with contributions from KW and FN. AS, FN, VB, and AK wrote the manuscript. All authors contributed to results interpretation and final manuscript preparation.
